# Perivascular epithelioid cell tumor of the uterine cervix identified on the liquid-based cytology: a case report

**DOI:** 10.1186/s13000-023-01290-3

**Published:** 2023-01-16

**Authors:** Xiao Tang, Min Feng, Yangmei Shen, Qijun Chen

**Affiliations:** 1 Department of Pathology, West China Second University Hospital, Sichuan University, No.20, Section 3, South Renmin Road, Chengdu, 610041 China; 2 Key Laboratory of Birth Defects and Related Diseases of Women and Children (Sichuan University), Ministry of Education, Chengdu, China

**Keywords:** Perivascular epithelioid cell tumor, Uterine cervix, Liquid-based cytology

## Abstract

**Background:**

Perivascular epithelioid cell tumor (PEComa) occurring in the female genital tract are rare, and typically found in the uterine corpus. PEComa occurring in the cervix is extremely rare, and very few cases have been reported till now. Cytological diagnosis of cervical PEComa is even rarer. So far, only two cases of PEComa diagnosed by conventional cervical smears have been reported.

**Case presentation:**

A 55-year-old postmenopausal woman presented with abnormal vagina discharge for 3 months. A liquid-based cytology test was performed. Microscopically, some loosely cohesive epithelioid cells were uniform with abundant clear cytoplasm, showing predominantly round or oval nuclei with finely stippled chromatin. Distinct round nucleoli were visible in some cells, notably with numerous melanin pigments in the cytoplasm. The cytopathological features were well correlated with cell block and histopathological findings. Upon immunohistochemistry (IHC), the tumor cells were positive for HMB45 and TFE3, focally positive for MelanA, while negative for muscle marker. Fluorescence in situ hybridization (FISH) confirmed *TFE3* gene rearrangement. The final pathological diagnosis was PEComa identified by the liquid-based cytology, cell block, cervical biopsy, IHC and FISH result. The patient underwent a total hysterectomy with bilateral salpingo-oophorectomy and was followed up for 2 years with no evidence of disease.

**Conclusion:**

The cytologic characteristics of the tumor can provide sufficient clues for PEComa diagnosis, which includes loosely cohesive, epithelioid morphology with abundant clear or eosinophilic cytoplasm, low-grade nuclear atypia, cytoplasmic melanin pigments. This will help cytopathologists to recognize this rare tumor that occurred in the cervix, and the combination of predictive morphology evaluation, immunophenotype, and molecular testing can achieve the definitive diagnosis of PEComa.

## Background

Perivascular epithelioid cell tumors (PEComas) are a rare group of mesenchymal neoplasms characterized by the presence of histologically and immunohistochemically distinctive perivascular epithelioid cells [1], which can occur in any part of the body. PEComa of the gynecological tract is very rare. It is most commonly encountered in the uterus corpus and very occasionally in the cervix, and only a few cases have been reported till now [2]. Cytological diagnosis of cervical PEComa is even rarer. So far, only two cases of PEComa diagnosed by conventional cervical smears have been reported in the available English literature [3, 4]. We report a case of cervix PEComa in a 55-year-old postmenopausal woman, initially identified by the liquid-based cytology test and subsequently confirmed by cell block, cervical biopsy, IHC and FISH result. Our finding indicated that a definitive diagnosis of PEComa can be rendered based on cytologic examination alone.

## Case presentation

**Fig. 1:**
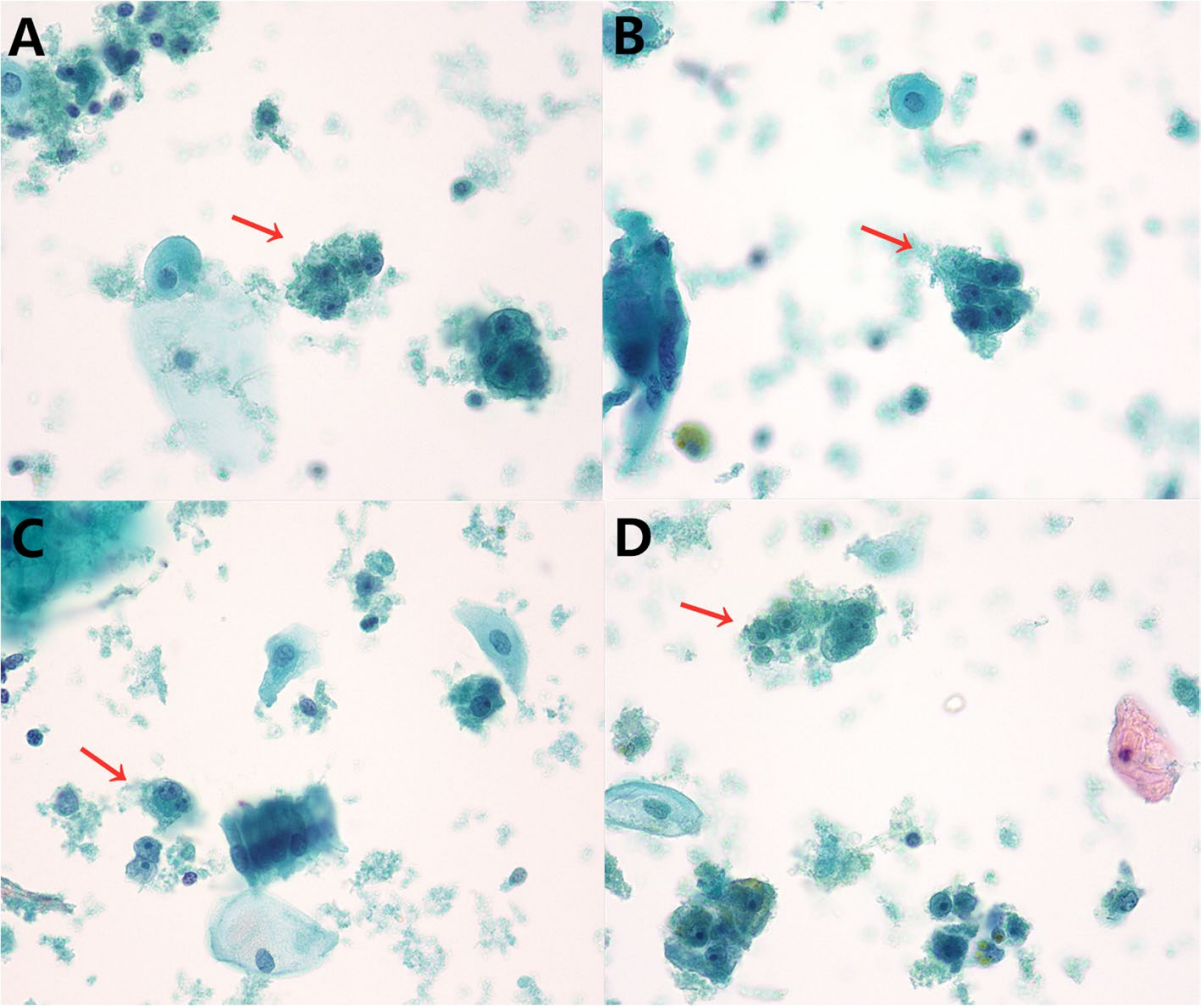
The epithelioid cells (red arrow) were uniform and approximately the same size as the parabasal cells, which with abundant clear cytoplasm (**A**&**B** Papanicolaou stain, × 400), showing predominantly round or oval nuclei with finely stippled chromatin. Distinct round nucleoli were visible (**C** Papanicolaou stain, × 400), numerous melanin pigments were identified in the cytoplasm (**D** Papanicolaou stain, × 400)

**Fig. 2:**
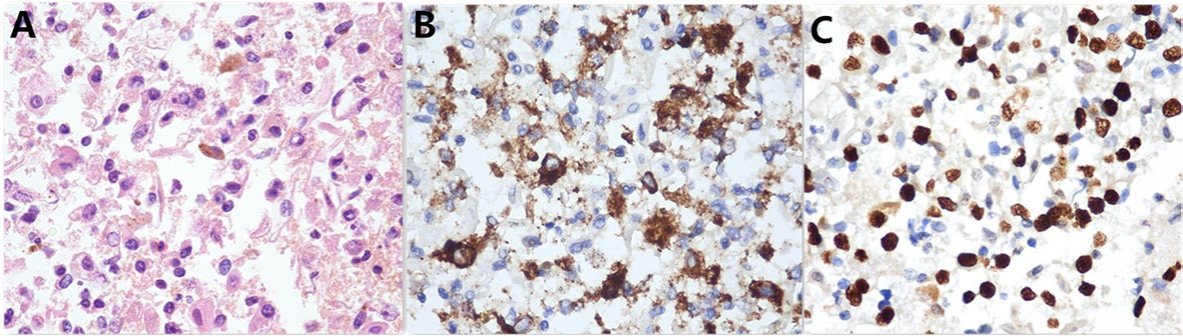
Cell block showed the epithelioid cells in a background of fibrinoid fluid, with abundant clear or granular eosinophilic cytoplasm (**A** H&E, × 400). IHC demonstrated that the epithelioid cells were positive for HMB45 (**B**) and TFE3 (**C**)

**Fig. 3:**
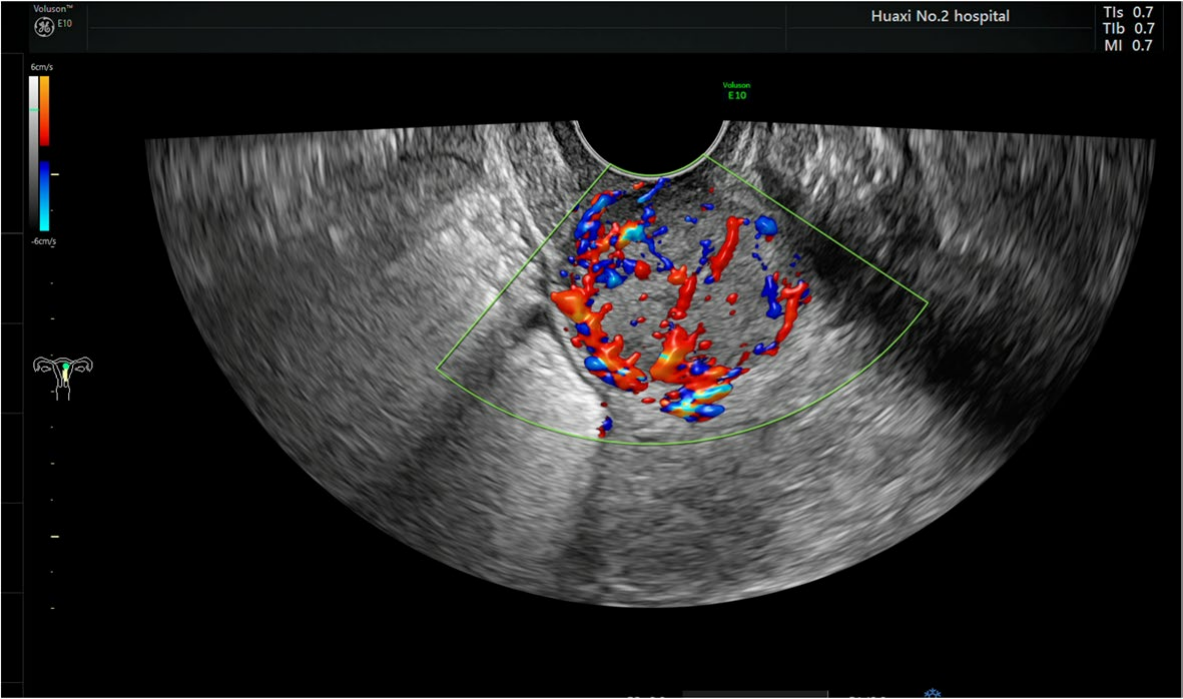
Transvaginal ultrasound examination showed an echogenic mass measuring 3 cm × 2.6 cm × 2.7 cm in the cervix with increased vascularity

**Fig. 4:**
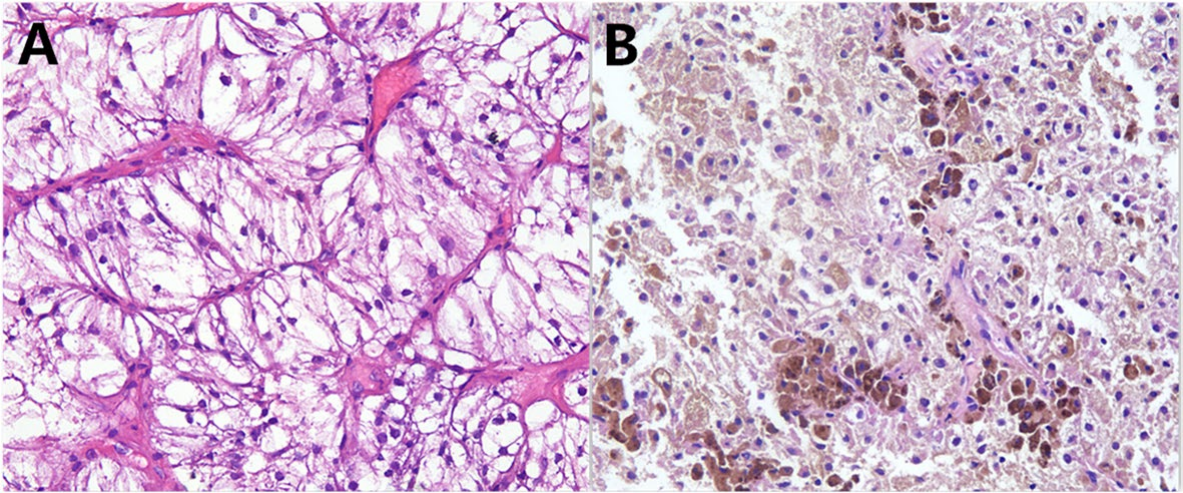
The epithelioid cells revealed clear or granular eosinophilic cytoplasm with central round to oval nuclei (**A** H&E, × 200), numerous melanin pigments were seen (**B** H&E, × 200). These two images are from cervical biopsy

**Fig. 5:**
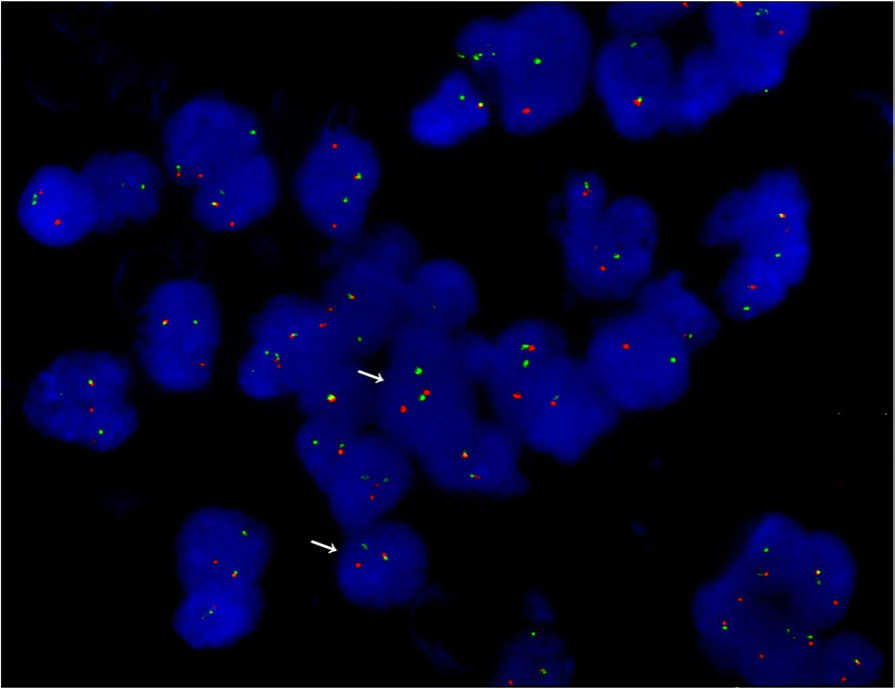
TFE3 break-apart fluorescence in situ hybridization showed one pair of split red and green signals (white arrow) and one fusion signals (two adjacent or overlapping red and green signals)

A 55-year-old woman presented with abnormal vagina discharge for 3 months after 4 years of menopause. Then, a liquid-based cytology test (BD Prep) was performed. Microscopically, we observed some loosely cohesive atypical cells arranged in single or clusters and sheets, which exhibited epithelioid morphology with abundant clear cytoplasm. The epithelioid cells were uniform and approximately the same size as the parabasal cells (Fig. 1A&B), showing predominantly round or oval nuclei with finely stippled chromatin. Distinct round nucleoli were visible in some cells (Fig. 1C), notably with numerous melanin pigments in the cytoplasm (Fig. 1D). The primary diagnosis was atypical cells which were suspected to be melanoma. Then we made a cell block from the remaining specimens. The cell block section showed single or clusters of medium-sized epithelioid cells in a background of fibrinoid fluid, with abundant clear or granular eosinophilic cytoplasm. Prominent nucleoli can be observed in some cells. A few spindled nuclei and melanin pigments were also identified (Fig. 2A). IHC demonstrated that the epithelioid cells were positive for HMB45 andTFE3 (Fig. 2B&C), focally positive for Melan-A, while negative for S-100, SOX-10, AE1/AE3, EMA, Desmin, SMA, H-caldesmon. Finally, a tentative diagnosis of PEComa was made by combining cell block and IHC, and with the statement that the final diagnosis will require more representative material. Subsequently, the transvaginal ultrasound examination showed an echogenic mass measuring 3 cm × 2.6 cm × 2.7 cm in the cervix with increased vascularity (Fig. 3), the patient underwent a colposcopic biopsy, which further confirmed our diagnosis. The epithelioid cells demonstrated clear or granular eosinophilic cytoplasm with central round to oval nuclei (Fig. 4A), radially arranged around blood vessels, and displayed no nuclear atypia and sparse mitotic activity. Numerous melanin pigments were observed (Fig. 4B). Immunohistochemical expression was also consistent with previous cell block. Additionally, Fluorescence in situ hybridization (FISH) confirmed *TFE3* gene rearrangement with the finding of split signal in tumor cells’ nuclei (Fig. 5). The final pathological diagnosis was PEComa. The patient underwent a total hysterectomy with bilateral salpingo-oophorectomy and was followed up for 2 years with no evidence of disease.

## Discussion

PEComas have been defined by the World Health Organization as a member of a family of mesenchymal neoplasms composed of perivascular epithelioid cells (PECs) that express melanocytic and smooth muscle markers [5]. PEComas arise most frequently in the uterine corpus and less commonly in the cervix. Moreover, the diagnosis of PEcoma by cervical cytology is much rarer. So far, only two cases of cervix PEComa diagnosed by conventional cervical smear have been reported. Our case is the first reported cervix PEComa diagnosed by a liquid-based cytology test. Compared with conventional cervical smear, liquid-based cytology test has the characteristics of clear background, distinct cell structure, and uniform distribution. It is difficult to diagnose based on a conventional smear alone. Since the characteristic perivascular arrangement cannot be discerned in cytologic smears, the application of a panel of immunocytochemical markers on cell blocks is likely to suggest a diagnosis of PEComa [6], especially in unusual sites like the cervix. Fortunately, in our case, the cytological features were consistent with the later biopsy.

The cytologic features of this case are similar to those reported in the previous studies, but there are also some differences. The similarities are that the tumor cells have an epithelioid morphology, appear relatively uniform and discohesive, and have characteristically abundant clear or granular eosinophilic cytoplasm. The difference is that our case contains a large number of melanin pigments in the cytoplasm, which is easily confused with melanoma. In addition, previously reported spindle cells or multinucleated giant cells are not found in our case. In previously reported two cases, one was initially diagnosed as a possible high-grade glandular lesion with a more specific diagnosis of clear cell carcinoma, and the other was primarily diagnosed with low-grade sarcoma. The final diagnosis of all cervix PEComa were based on the result of the biopsy. In our case, the tumor was initially considered as a melanoma because of many melanin pigments in the cytoplasm. The uterine sarcomas were excluded in the differential diagnosis because of the absence of spindle cell components. Combined with the results of cell block, cervical biopsy, IHC, and FISH result, the final diagnosis of PEComa was made.

The differential diagnoses by cervical cytology include many lesions. Firstly, melanoma, in whichtumor cells are typically pleomorphic, with discrete distribution ranging from round, oval, and spindle-shaped, containing large nuclei, coarsely clumped, irregularly distributed chromatin prominent nucleoli. Additionally, binucleation and intranuclear pseudo-inclusions may be identified. The cytoplasm is well-defined with or without cytoplasmic melanin pigments. The background is dirty, necrotic, inflammatory, or hemorrhagic because of tumor diathesis [7]. IHC can be useful for differentiating these lesions. PEComa is positive for myoid markers and negative or focally positive for S-100 protein expression in contrast to melanoma [5]. Controversial cases can be identified by FISH. Secondly, reactive endocervical cells should be excluded. In whichthe nuclei of reactive cervical glandular cells show a variable increase in nuclear size, with prominent nucleoli and uniform finely stippled chromatin. Moreover, they are usually round and plump, not as loosely cohesive as PEComa cells. Rare intracytoplasmic polymorphonuclear leukocytes are seen, although they are a worrisome feature of endometroid endometrial carcinoma; Thirdly, clear cell carcinoma (CCC), cytologically, there are some overlaps between CCC and PEComa, but the nuclear pleomorphism of CCC is striking. It often contains prominent nuclei that can be hyperchromatic and pleomorphic and project into the glandular lumen to form hobnail cells. On IHC, CCC expresses epithelial markers (AE1/AE3 and EMA), and does not show the “myomelanocytic” phenotype of PEComa. Fourthly, endometrial or ovarian adenocarcinoma, three-dimensional groups and clusters or papillary configurations are more common in endometrial or ovarian adenocarcinoma cells, and the nuclear atypia is more obvious, including nuclear hyperchromasia and pleomorphism. These features are uncommon in PEComa. Fifthly, alveolar soft part sarcoma (ASPS) is also rare in the cervix, but the cytological morphology and immunohistochemical features of PEComa and ASPS can sometimes be similar. Some ASPS also express TFE3 [8]. However, ASPS exhibits smooth muscle markers, such as SMA but is invariably negative for melanocytes markers [9].

Although most PEComas harbor loss-of-function TSC1/TSC2 mutations, a small subset of PEComas show rearrangement of the *TFE3* gene [10]. Recently, it was suggested that *TFE3* translocation-associated PEComas of the gynecologic tract represent a distinct form of this tumor. Morphological features of these tumors include alveolar or nested growth, predominant epithelioid component, low nuclear atypia, and rare mitoses, IHC showed strong expression of HMB45 and TFE3, focal or absent for MelanA and smooth muscle markers [8]. In our case, FISH confirmed *TFE3* gene rearrangement. Both morphology and immunophenotype of the tumor were consistent with those *TFE3* translocation-associated PEComas described previously.

Because of the rarity of cervix PEComa, there are no standardized guidelines for treatment. Complete surgical resection with a tumor-free margin is usually considered to be the standard treatment [11]. Chemotherapy and radiotherapy have not yielded conclusive results [10]. Some histologic features were associated with the aggressive behavior of uterine PEComa, including tumor size > 5 cm, high nuclear grade, >1 mitosis/50 HPF, necrosis, and vascular invasion. All of these were absent in our case. The tumor displayed no evidence of malignancy. The patient underwent total hysterectomy with bilateral salpingo-oophorectomy and was followed up for 2 years without any evidence of disease progression.

In conclusion，this is the first case of cervix PEComa identified on the liquid-based cytology. The cytologic characteristics of the tumor can provide sufficient clues for diagnosing a PEComa, including loosely cohesive, epithelioid morphology with abundant clear or eosinophilic cytoplasm, low-grade nuclear atypia, and cytoplasmic melanin pigments. The pathologist should be familiar with these cytological features, and the combination of histological biopsy, immunophenotype, and molecular testing, can achieve the definitive diagnosis of PEComa.

## Data Availability

All data generated or analyzed during this case are included within the article.

## Abbreviations

PEComas: Perivascular epithelioid cell tumours
IHC: Immunohistochemistry
FISH: Fluorescence in situ hybridization
CCC: Clear cell carcinoma
ASPS: Alveolar soft part sarcoma
